# Fluorimetric Detection of Single Pathogenic Bacterium in Milk and Sewage Water Using pH-Sensitive Fluorescent Carbon Dots and MALDI-TOF MS

**DOI:** 10.3390/microorganisms8010053

**Published:** 2019-12-26

**Authors:** Qiaoli Yang, Umer Farooq, Wei Chen, Muhammad Wajid Ullah, Shenqi Wang

**Affiliations:** 1 Advanced Biomaterials & Tissue Engineering Centre, College of Life Science and Technology, Huazhong University of Science and Technology, Wuhan 430074, China; D201677468@hust.edu.cn (Q.Y.); uf.wazir786@yahoo.com (U.F.); 2 Department of Biomedical Engineering, Huazhong University of Science and Technology, Wuhan 430074, Chinawajid_kundi@yahoo.com (M.W.U.)

**Keywords:** carbon dots, pH-sensitivity, fluorimetry, *E. coli* O157:H7 detection, MALDI-TOF MS

## Abstract

The current study is focused on the application of water-soluble, fluorescent, and pH-sensitive carbon dots (CDs) as a nanoprobe for sensitive detection of pathogenic bacteria in milk and sewage water. The CDs were facilely synthesized through the controlled carbonization of sucrose using sulfuric acid and characterized through XRD, FTIR, TEM, UV-Vis Spectroscopy, and fluorescent analysis. The as-synthesized CDs were highly water-soluble, stable, and pH-sensitive fluorescent nanomaterials. The pH-related fluorescence study showed that the ratio of fluorescence intensity (Log[I_F410_/I_F350_]) changed linearly in the pH range between 4.9 and 6.9 in the Britton-Robison buffer. By determining the pH variation of the growth medium caused by the released acidic metabolites, the CDs-based ratiometric nanoprobe and MALDI-TOF mass spectrometry (MS) were used for the detection and identification of *Escherichia coli* O157:H7, respectively. The practical applicability of the pH-sensitive fluorescent CDs-based ratiometric nanoprobe was evaluated to detect *Escherichia coli* O157:H7 in real samples, i.e., milk and sewage water using agar count plate method with a limit of detection (LOD) up to 1 colony-forming unit per mL (CFU/mL).

## 1. Introduction

*Escherichia coli* O157:H7 (*E. coli* O157:H7) is a common enterohemorrhagic bacteria and the main causative agent of food and water-borne diseases [1]. It is responsible for many foodborne outbreaks globally, which cause serious problems to public health and the economy [2]. The culture-reliant conventional *E. coli* O157:H7 detection approach is laborious, suffering from the interference of complex food matrices, and time-consuming, taking 1–3 days [3,4], and needing skilled operators [5]. To overcome such limitations, polymerase chain reaction (PCR) [6] has been developed but this method used for *E. coli* O157:H7 detection also needs a detection time of about 24 h [7]. Several bioanalytical techniques have been developed over the last few years, like surface-enhanced raman spectroscopy (SERS) [8], flow cytometry [9], fluorescent methods [10], lateral flow immunoassay [11], hybridization chain reaction (HCR) [12], and amperometric immune sensors [13]. Among these approaches, the fluorescent technique has drawn a great deal of attention from researchers owing to its outstanding selectivity, extraordinary sensitivity, cost-effectiveness, and is non-disparaging [14]. To date, different types of fluorescent nanomaterials and organic dyes have been reported for *E. coli* O157:H7 detection. For instance, dye-doped fluorescent silica nanoparticles composite [15], CdTe/CdS quantum dots (QDs) [16], time-resolved fluorescent nanobeads (TRFN) [17], fluorescent microspheres (FM) [18], and aggregation-induced emission (AIE)-based materials [19] have been reported for *E. coli* O157:H7 sensing. However, the complex synthesis procedures of fluorescent materials as well as the cytotoxic effects of some fluorescent materials such as heavy metal-based (e.g., Pb, Cd, Hg) QDs restrict their practical applications in bacterial detection [20]. For example, stained silica nanoparticles have a high affinity to discharge some of the trapped fluorophores; however, their photo-bleaching effect prevents their long-term applications in vivo [21]. Similarly, the inorganic hybrid nanomaterials, such as QDs [22] or lanthanide-loaded silica nanoparticles [23], are photo-stable substitutes as compared to the stained nanoparticles; however, the range of their in vivo practical applications remain narrow due to their tedious and multistep synthesis procedures as well as concerns related to their toxicity [24].

At present, carbon dots (CDs) have gained significant consideration owing to their characteristic properties like low cytotoxicity, high chemical stability, water solubility, and lack of blinking [25]. CDs are synthesized by several methods, including both bottom-up (e.g., hydrothermal carbonization and thermal decomposition) and top-down (e.g., chemical oxidation and electrochemical exfoliation) [26]. Most of the reported techniques used for the synthesis of CDs did not receive practical application due to their complex synthesis procedures and the requirements for costly apparatus [25]. Additionally, the reported CDs mostly required further modification and passivation to impart various functional groups [27].

Bacterial cells, like *E. coli*, produce and release various acidic metabolites such as lactic acid, acetic acid, and CO_2_, etc. in the medium that decreases the pH of the growth medium [28]. This variation in pH provides the base for the development of pH-based methods for the detection of bacteria [29]. However, the durability of most pH-based fluorescent methods is limited due to their photo-bleaching effect and inconsistency in the complex medium [30]. Thus, pH-based detection methods require highly stable fluorescent nanomaterials that can retain fluorescent properties in a complex environment for the detection of pathogenic bacterial strains. In addition, such nanomaterials should be cheap, non-toxic, and can be facilely produced to develop an artless, cheap, and efficient technique for bacterial detection. On the other hand, matrix-assisted laser desorption ionization-time of flight mass spectrometry (MALDI-TOF MS) is a rapid, accurate, and reliable approach for bacterial identification as compared to the conventional phenotypic techniques or molecular methods [31].

**Scheme 1:**
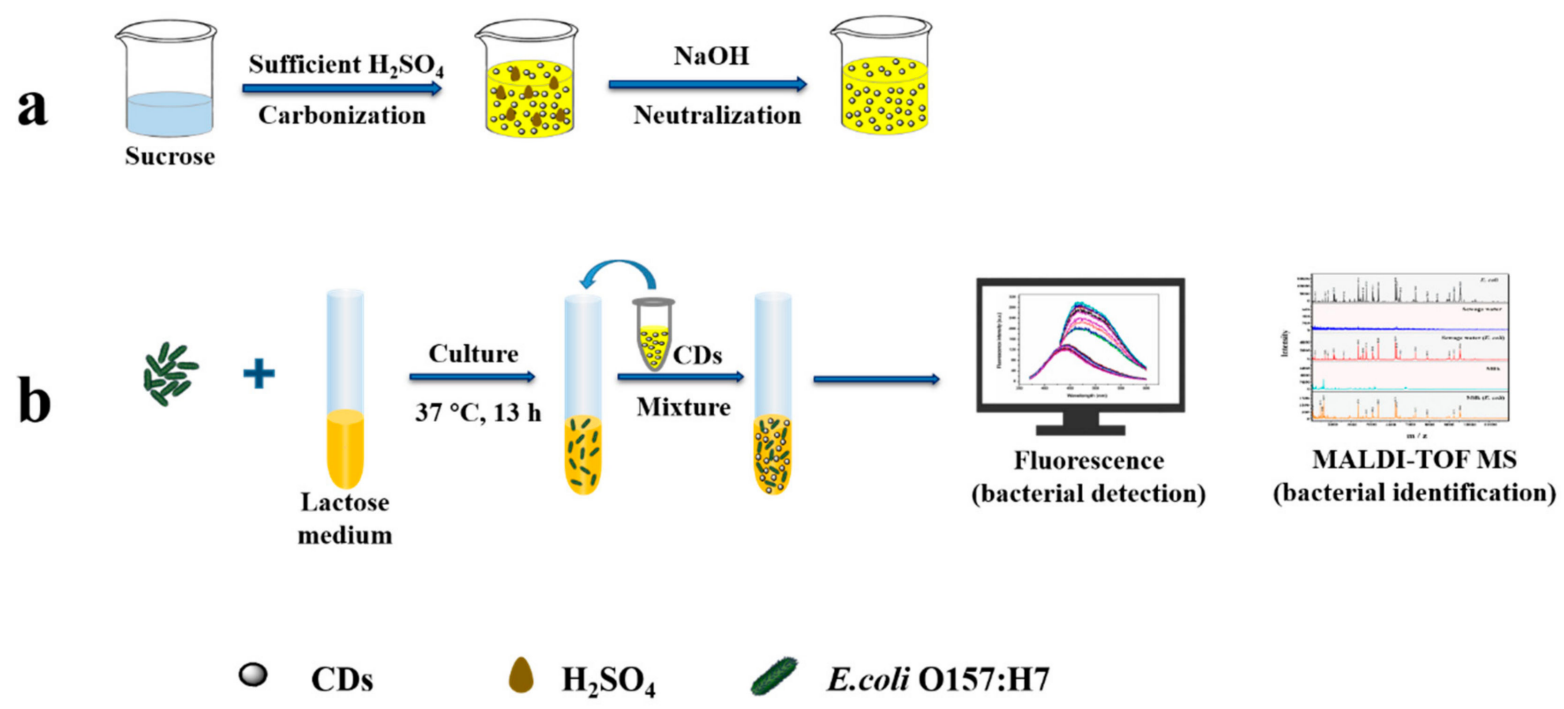
Schematic representation of (**a**) carbon dots (CDs) solution synthesis through carbonization of sucrose with sulfuric acid and (**b**) application of the pH-sensitive fluorescent CDs to fluorimetrically detect *E. coli* O157:H7 with validation by MALDI-TOF MS.

The current study is aimed to fluorimetrically detect pathogenic bacteria by using the pH-sensitive and fluorescent CDs, and MALDI-TOF MS (Scheme 1). The water-soluble, stable, low-cost, and pH-sensitive fluorescent CDs were facilely synthesized through carbonization of sucrose with sulfuric acid and characterized for various properties. The as-synthesized CDs effectively detected microbial cells in milk and sewage water while MALDI-TOF MS confirmed the bacterial strains. Recently, Bhaisare et al. reported magnetic CDs involving fluorimetric detection followed by MALDI-TOF MS for the detection of *E. coli* [32]. The reported LOD for *E. coli* was 3.5 × 10^2^ CFU/mL. Comparatively, LOD of 1 CFU/mL of *E. coli* in milk and sewage water is reported in the present study, by application of the simply synthesized CDs involving fluorimetric detection followed by MALDI-TOF MS. The developed fluorimetric detection method using a CDs-based ratiometric pH probe can find potential industrial and medical applications for the detection of unwanted and pathogenic bacteria.

## 2. Results

### 2.1. Characterization of CDs

**Figure 1:**
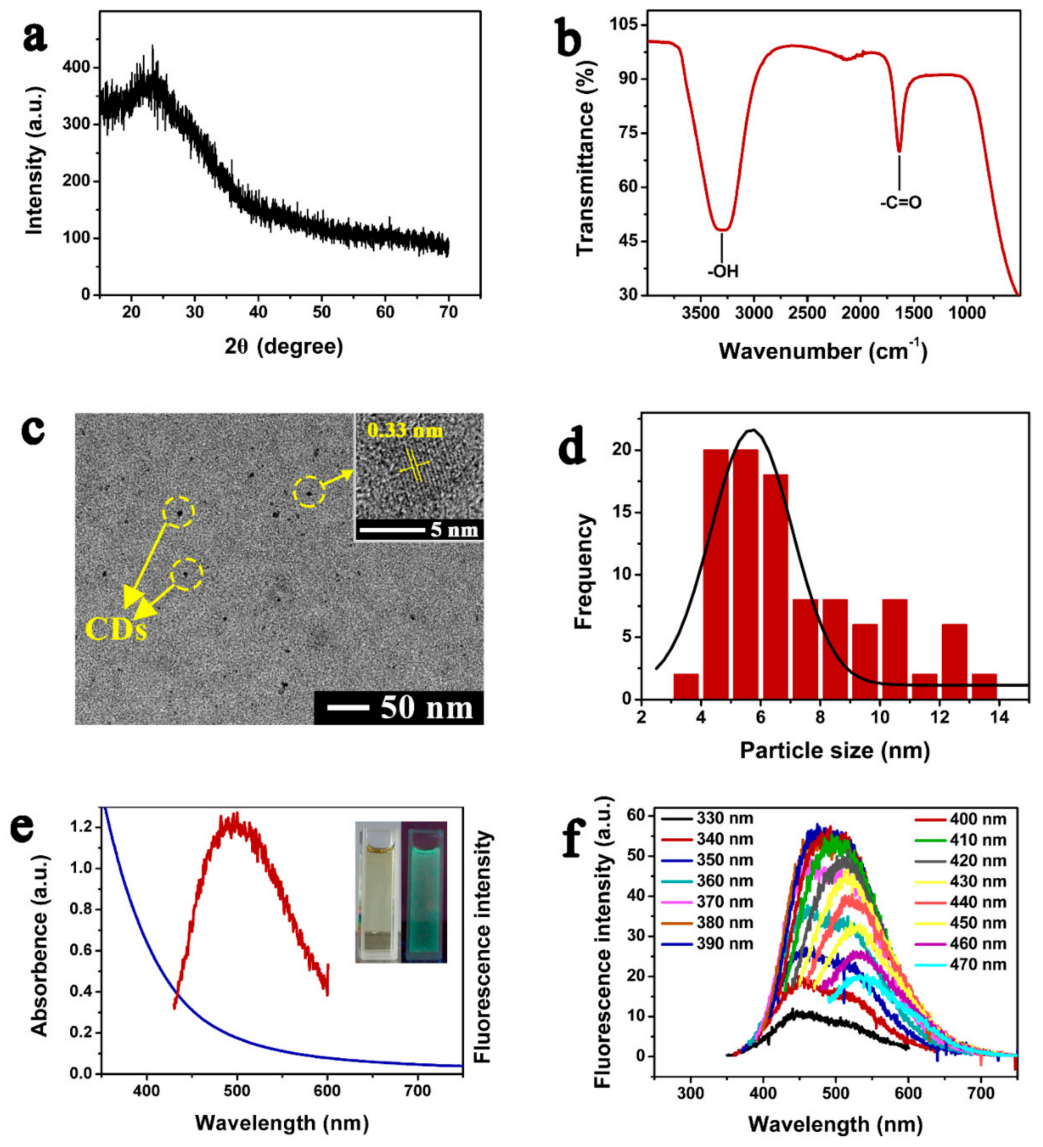
Characterization of CDs. (**a**) X-ray powder diffraction pattern; (**b**) functional groups analysis by FTIR; (**c**) TEM micrograph; (**d**) particle size distribution for 100 randomly selected particles; (**e**) absorption and emission spectra of the fluorescent CDs. Inset: the optical image of CDs solution in natural light (left) and UV light (right); (**f**) CDs emission spectra at different excitation wavelengths, with a gradual increase from 330 nm to 470 nm (with increments of 10 nm).

The fluorescent CDs solution was synthesized by successive carbonization of sucrose as reported [33] and optimized fluorescently under variable pH values during the synthesis (Figure S1). The XRD pattern of hydrophilic CDs showed a broad peak at 2θ = 20~23° (Figure 1a). The FTIR spectrum of as-synthesized CDs (Figure 1b) showed a broad peak at 3309 cm^−1^ and a small sharp band at 1635 cm^−1^, assigned to the –OH stretching vibration and –C=O stretching vibration. The TEM micrographs (Figure 1c) showed that the synthesized CDs were spherical in shape. The inset in Figure 1c shows the high-resolution TEM image of the CDs, which displays that the distance between the lattice fringes is 0.33 nm. The particle size of CDs was estimated by randomly selecting 100 particles from the TEM micrograph (Figure 1c) and represented as a distribution curve (Figure 1d). The distribution curve of CDs particle size showed the average particle size of the synthesized fluorescent CDs was 7.2 ± 2.6 nm.

The UV-Vis absorption and fluorescence emission spectra of the synthesized fluorescent CDs dispersed in water are shown in Figure 1e. The absorption spectrum of CDs didn’t show a clear band (Figure 1e). Additionally, a maximum fluorescence emission peak was observed at 500 nm when CDs were excited at 410 nm (Figure 1e). The inset in Figure 1e showed that the color of the CDs solution was transparent pale yellow and green in natural and UV lights, respectively. To further investigate the optical properties of synthesized CDs, the detailed fluorescence emission spectra were determined at different excitation wavelengths (Figure 1f). The results showed that the emission wavelength was shifted from 450 to 550 nm when the excitation wavelength was increased from 330 to 470 nm. Furthermore, the fluorescence intensity was first gradually increased, followed by a gradual decrease.

### 2.2. pH-Dependent Fluorescence Performance and Stability of CDs in Britton-Robison Buffer

**Figure 2:**
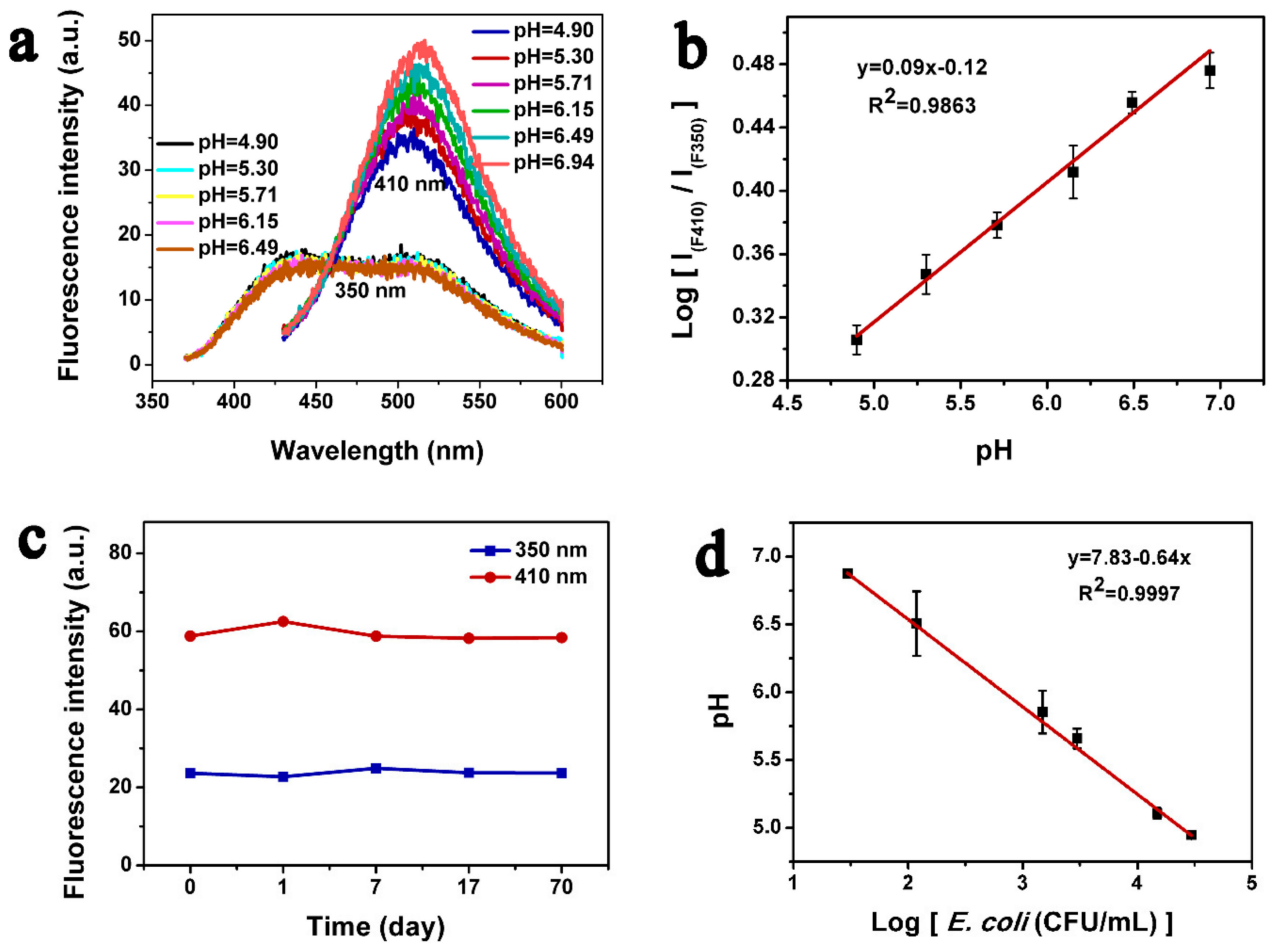
The fluorescent features of CDs. (**a**) Fluorescent spectra of CDs in a Britton-Robison buffer with different pH values; (**b**) ratios of fluorescence [I_F410_/I_F350_] vs. various pH values in the Britton-Robison buffer. The standard deviation from triplicate experiments is represented by error bars; (**c**) fluorescence stability observation of CDs through a fluorescence spectrophotometer at excitation wavelengths of 350 nm and 410 nm; (**d**) Variation in pH of lactose-selective medium inoculated with *E. coli* O157:H7 plotted against bacterial cell density. The pH of the medium was measured via pH-meter, while the density of viable bacterial cells was determined by colony count method. The standard deviation from the triplicate experiments is represented by error bars.

When CDs were added in different pH of Britton-Robison buffers, the correlation of pH values and the fluorescence intensity was investigated (Figure 2a). When excited at a shorter wavelength (350 nm), the fluorescence intensity was decreased with the increasing pH value. In contrast, the fluorescence intensity was increased with the increasing pH value when excited at a longer wavelength (410 nm). Furthermore, the pH value and the fluorescence intensities (Log[I_F410_/I_F350_]) were plotted at excitation wavelengths of 410 nm and 350 nm, and the results are shown in Figure 2b. The results showed a linear trend in the pH range of 4.9 to 6.9 in the Britton-Robison buffer. The fluorescence stability of synthesized CDs, stored at 4 °C, was determined at different time intervals. The results showed no obvious decrease in fluorescent intensity within 70 days when excited at 350 nm and 410 nm (Figure 2c).

### 2.3. The Effect of Bacterial Metabolism on pH

The bacterial cells were grown in a lactose-selective medium at 37 °C for 13 h. Then the pH of the bacterial culture was determined via pH-meter, while the bacterial cell density was determined by the agar plate count method. The results showed an inverse relationship between the bacterial cell density and pH of the culture in the range between 29 and 2.9 × 10^4^ CFU/mL of bacteria (Figure 2d), and the linear regression equation is y = 7.83 − 0.64x and R^2^ = 0.99. The pH at a minimum of 29 CFU/mL was found to be 6.8, while at a maximum of 2.9 × 10^4^ CFU/mL was found to be 4.9.

### 2.4. Bacterial Detection with CDs-Based Ratiometric pH Probe

**Figure 3:**
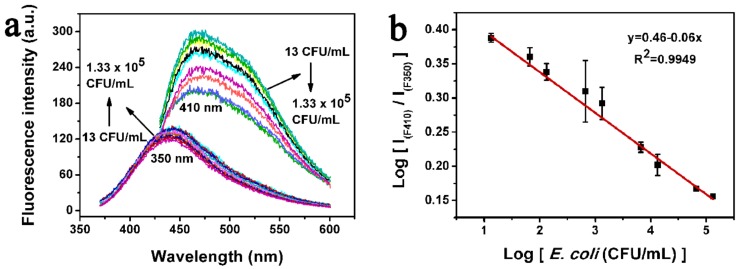
The relationship between *E. coli* O157:H7 cell density and fluorescence intensity of CDs. (**a**) The fluorescence intensity of mixture changed with the different densities of *E. coli* O157:H7 in CFU/mL, at excitation wavelengths of 350 nm and 410 nm; (**b**) The graph of the logarithm of *E. coli* O157:H7 cell density drawn against the ratio of fluorescence intensity [I_F410_/I_F350_] showed a linear correlation in the density range of 13 to 1.33 × 10^5^ CFU/mL. The standard deviation from triplicate experiments is represented by error bars.

The bacterial cells were cultured in a lactose-selective medium for different cell densities. After the addition of fluorescent pH-sensitive CDs into the bacterial culture, the fluorescent intensity of the mixture at two different emission wavelengths (410/350 nm) was measured. The volume of CDs added in the bacterial solution was optimized and 1 mL CDs solution was recorded to be optimum (Figure S2). The fluorescence intensity of the mixture increased with the increasing bacterial cell density at an excitation wavelength of 350 nm; however, a decrease in fluorescent intensity was recorded with the increasing bacterial cell density at an excitation wavelength of 410 nm (Figure 3a). The cell density of *E. coli* O157:H7 culture showed a linear relationship with the ratio of fluorescence intensity [I_F410_/I_F350_] in the range of 13 to 1.33 × 10^5^ CFU/mL of bacteria (Figure 3b), while the linear regression equation was y = 0.46 − 0.06x, and R^2^ = 0.99 at the limit of detection (LOD), at an S/N ratio of 3, was found to be 1 CFU/mL.

### 2.5. Real Samples Detection

**Scheme 2:**
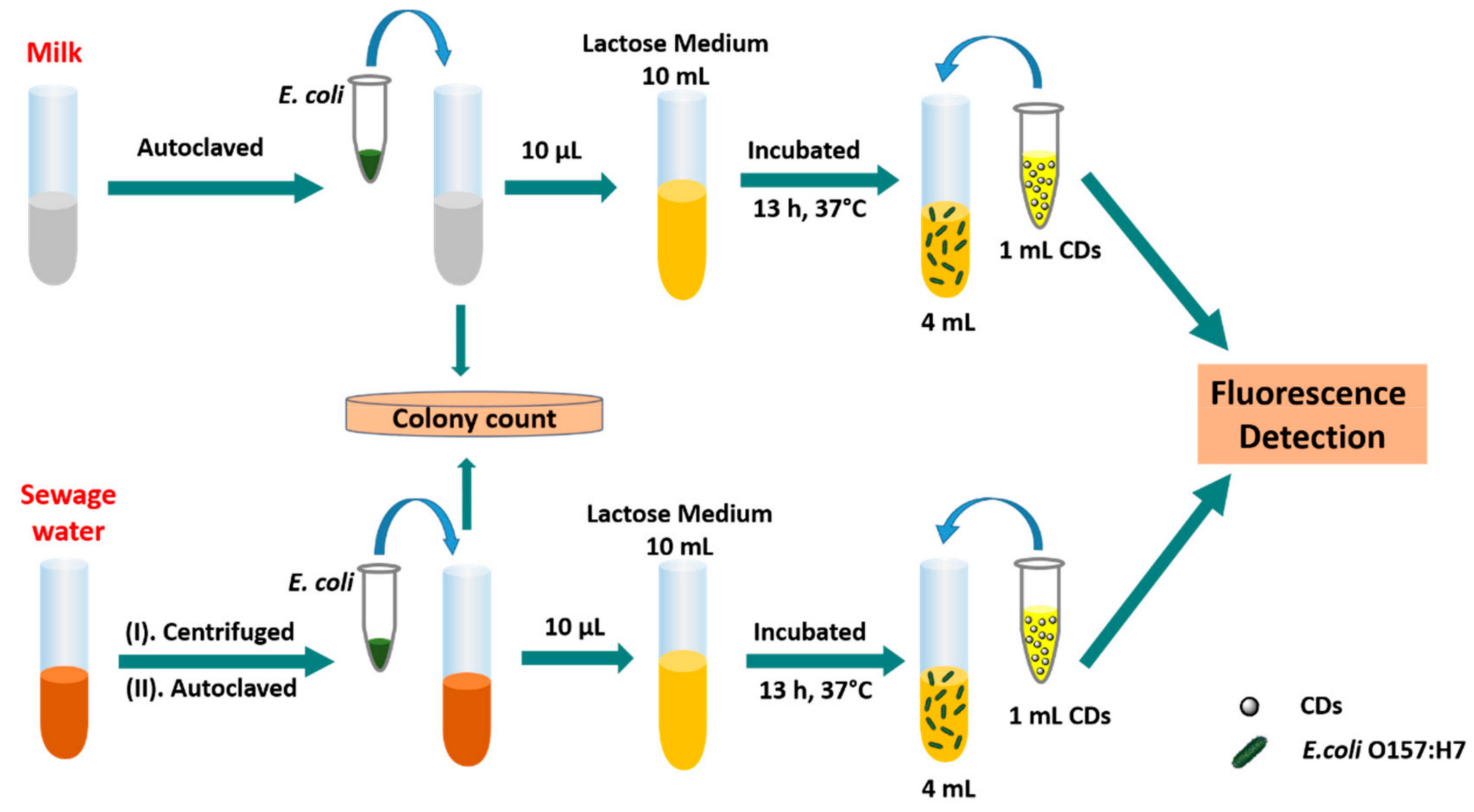
Schematic representation of CDs-based fluorescent detection of *E. coli* O157:H7 in the milk and sewage water. *E. coli* cells were inoculated into samples (milk and sewage water) and bacteria were enumerated by plate count method, followed by incubation in the lactose-selective medium. After 13 h incubation, CDs-based fluorescent detection was performed and the results were analyzed.

The potential application of synthesized CDs was determined by evaluating their bacterial detection ability in real samples, such as milk and sewage water (Scheme 2). For analysis, the desired amount of *E. coli* O157:H7 cell culture was inoculated into the milk and sewage water samples. Meanwhile, the bacterial cell densities were determined by using the plate count method. The real samples that inoculated with the bacterial cells were incubated in the lactose-selective medium. After incubation, the bacterial cells were detected in real samples by adding CDs followed by fluorescence detection, and the results are shown in Table 1.

### 2.6. The MALDI-TOF MS for Bacterial Identification

**Figure 4:**
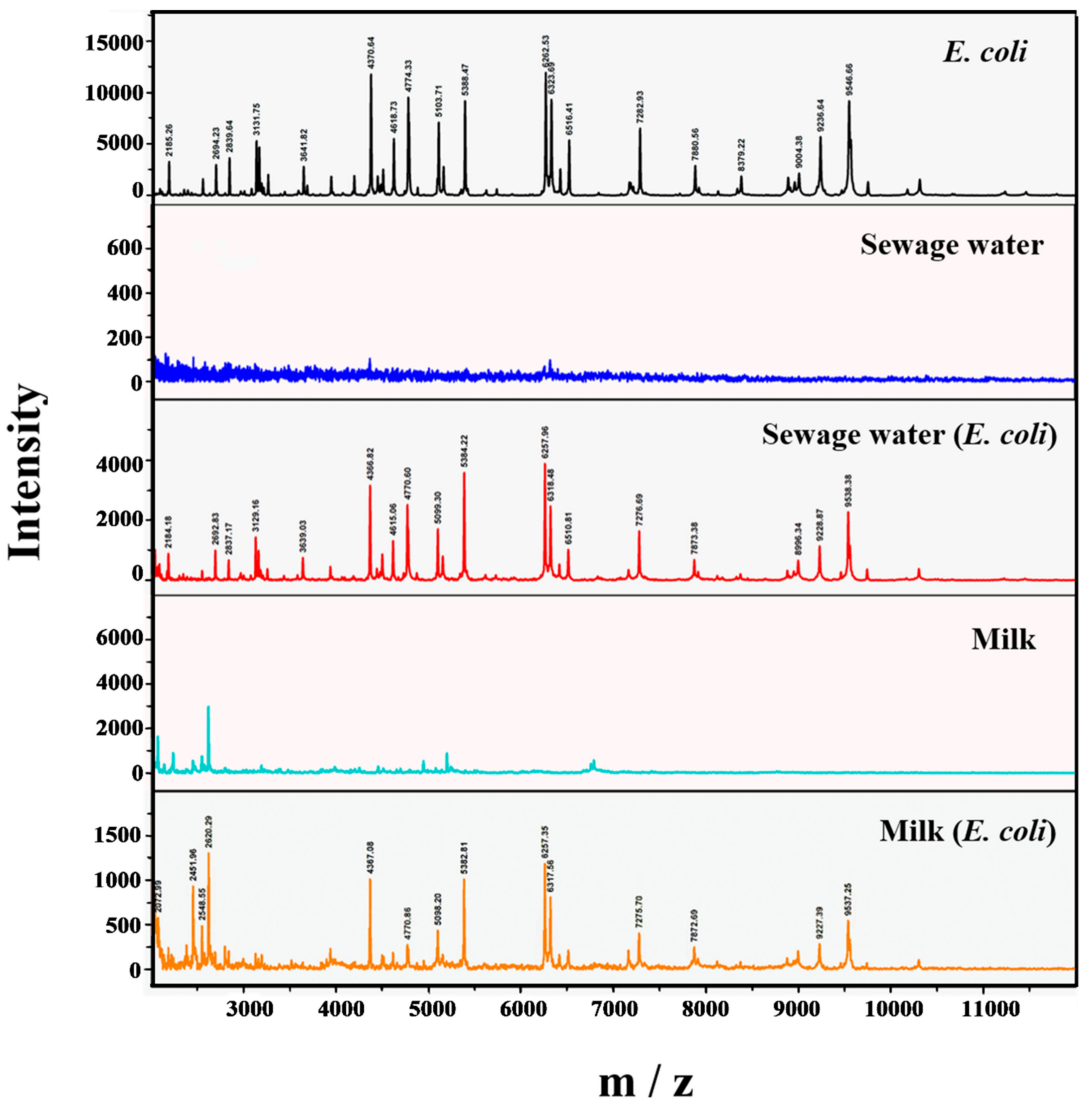
MALDI-TOF MS spectra of bacteria (positive control), sterilized sewage water (negative control), sterilized milk (negative control), sewage water inoculated with bacteria, and milk inoculated with bacteria. The characteristic peaks of *E. coli* O157:H7 and spectra of sterilized milk and sewage water samples are shown. The spectrum for the sewage water and milk spiked with bacteria (test samples) are comparable with the spectrum of both positive (*E. coli*) and negative controls (sterilized milk and sewage water).

The identification of bacteria through MALDI-TOF MS is carried out by comparing the ions and relative strength of the obtained peaks for unknown samples with the super and reference spectra in the standard spectrum in the database. The MALDI-TOF MS spectra of the bacteria-treated milk and sewage water samples, as well as sterilized milk and sewage water samples, are shown in Figure 4. The characteristic peaks at m/z 4370.64, 4774.33, 5103.71, 5388.47, 6262.53, 7282.93, 7880.56, and 9546.66 were observed in the spectrum of *E. coli* control. The background spectra of the sterilized milk and sewage water were also collected, which did not show any specific signal in the target spectral range. The additional peaks in the spectral range 2000–2600 m/z in the milk sample could be due to the presence of different proteins in the milk sample when compared with the standard *E. coli* spectrum. The spectrum for the sewage spiked with bacteria showed characteristic peaks at m/z 4366.82, 4770.60, 5099.30, 5384.22, 6257.96, 7276.69, 7873.38, and 9538.38, which are similar to the *E. coli* control. Similarly, the spectrum for milk spiked with bacteria characteristic peaks were at m/z 4367.08, 4770.86, 5098.20, 5382.81, 6257.35, 7275.70, 7872.69, and 9537.25, which were also comparable with the spectrum of *E. coli* control.

## 3. Discussion

The fluorescent CDs solution synthesized by successive carbonization of sucrose was characterized and optimized fluorescently by comparing the fluorescent intensity at variable pH values of CDs solutions (Figure S1, detailed in the Supplementary Materials). As shown in Figure 1a, the XRD analysis showed a broad peak that is consistent with the carbon crystal plane (002) and suggested the amorphous characteristics of carbon, as reported in the literature [34]. The FTIR spectrum (Figure 1b) reveal these oxygen-containing groups on the surface of CDs. They could be introduced by the concentrated sulfuric acid, imparting the hydrophilic properties to CDs [35]. The TEM micrographs (Figure 1c) revealed the spherical shape of CDs and the distance between the lattice fringes corresponded to the carbon crystal plane (002) [36], which is consistent with the XRD spectrum. In the results of UV-Vis absorption and fluorescence emission spectra of the synthesized fluorescent CDs (Figure 1e), the absorption spectrum of CDs did not show a clear band, which is in agreement with a previous report [37]. The detailed fluorescence emission spectra (Figure 1f) were determined at different excitation wavelengths, and the emission spectra of CDs showed excitation-dependency which is assumed generally that the quantum size, surface/edge, and molecular fluorophore are the key features contributing to the fluorescence effect [38]. Furthermore, the fluorescence intensity was first gradually increased, followed by a gradual decrease, which can be attributed to the different emission sites on the surface of CDs as well as their different sizes, as reported previously [39].

The fluorescent behavior of the CDs as demonstrated in Figure 2a further revealed that when excited at a wavelength of 350 nm, the fluorescence intensity of synthesized CDs was decreased with the increasing pH value; however, the fluorescence intensity was increased with the increasing pH value at an excitation wavelength of 410 nm. These variations in fluorescence performance of CDs could be attributed to the presence of different functional groups, such as –OH and –COOH, on their surface [40]. With the increasing pH, the –COOH groups are ionized gradually, which results in a variation of surface charge of CDs. At the same time, the electrostatic interaction between the –COOH group results in the dispersion of CDs that result in an enhanced fluorescence activity, thus confirming the pH-dependent fluorescence performance of CDs [40]. The correlation of various pH and the fluorescence intensity of CDs was studied (Figure 2b), revealing there is a linear correlation of various pH and the ratio of fluorescence intensity at the different excitation wavelength [I_F410_/I_F350_], suggesting a potential application for the development of a ratiometric pH probe. While comparing with the light-stability of previously reported CDs [37], our synthesized CDs showed fluorescence stability for a prolonged time at 4 °C (Figure 2c). The better fluorescence stability of the synthesized CDs suggests their application in bacterial detection and other fields.

The lactose uptake by bacterial cells cultured in the lactose-selective medium resulted in the release of acidic metabolites that fluctuated the pH of the medium [28]. The linear correlation between pH and bacterial cell density (Figure 2d) demonstrates that the pH of the bacterial culture could be used as an indicator for the detection of bacterial cells. In summary, the fluorescent density of CDs has linear influence with pH, and the bacterial cell density has linear correlation with the pH of culture. Relying on these principles, bacterial cell density can be fluorometrically detected by using pH-sensitive CDs.

Most materials which have pH-dependent fluorescence property were utilized for bacterial detection [41]. However, their fluorescent intensity at constant wavelength remained variable (increases/decreases) with the change of pH values, and furthermore, they were greatly affected by other factors of the system as stated elsewhere [42]. Alternatively, we developed a CDs-based ratiometric pH probe to measure the bacterial cell density by the ratio of fluorescent intensity at two different emission wavelengths (410/350 nm), which can eliminate the interference of other factors in the system by internal reference [43]. When excited at an excitation wavelength of 350 nm, the fluorescence intensity of the mixture increased along with the increasing bacterial cell density. However, the fluorescence intensity of the mixture decreased along with the increasing bacterial cell density at an excitation wavelength of 410 nm (Figure 3a). As discussed earlier, the key factors possibly contributing to the inverse fluorescence behavior of CDs are the different emission sites on the surface of CDs as well as their different sizes [39]. Another possible reason is the gradual ionization of –COOH groups on the surface of CDs under various pH which result in the dispersion of CDs and enhance fluorescence activity [40].

In general, the exclusive fluorescent properties of CDs put forward their potential application by measuring the pH sensitivity, and the variations in pH of the bacterial culture that could be used as an indicator for the detection of bacterial cells. Relying on these principles, bacterial cell density can be fluorometrically detected by applying the ratiometric CDs-based pH probe. As investigated in the results section (Figure 3b), the low LOD of 1 CFU/mL was attributed to ratiometric CDs-based probe and their high fluorescent sensitivity, generating a signal even at low *E. coli* O157:H7 concentration and further, the ratiometric CDs-based probe eliminated the interference of other factors in the system by internal reference. These results show that the CDs-based fluorescence bacterial detection offers a wide detection range at relatively high sensitivity and low limit of detection.

The application of synthesized CDs-based ratiometric pH probe was determined by evaluating their utilization for bacterial detection in milk and sewage water samples. The results suggest that a reliable, simple, and sensitive bacterial detection method was achieved by application of the synthesized CDs probe. Comparative efficiency of previously reported ratiometric pH probes and the CDs-based ratiometric pH probe in the present study is given in Table 2, indicating their superiority in terms of simple synthesis and improved LOD.

The identification of bacteria through MALDI-TOF MS is carried out by comparing the ions and relative strength of the obtained peaks for unknown samples with the super and reference spectra in the standard spectrum in the database [31]. As shown in Figure 4, the presence of standard peaks in the spectra of bacteria-treated milk and sewage water samples implies that MALDI-TOF MS effectively detected *E. coli* O157:H7 even in such a complex environment like sewage water and milk, thus validating the results of fluorimetric and pH-sensitive fluorescent carbon dots-based detection of *E. coli*.

## 4. Materials and Methods

### 4.1. Materials

The materials including sucrose, sulfuric acid (H_2_SO_4_), sodium hydroxide (NaOH), lactose, bovine bile salt, sodium carboxymethyl cellulose, sodium chloride, phosphoric acid, acetic acid, boric acid, and dipotassium phosphate were purchased from Sinopharm Chemical Reagent Co. Ltd., China (https://www.sinoreagent.com). Peptone was supplied by Shanghai Shengsi Biochemical Technology Co. Ltd., China (http://ssincere.com). Yeast extract was purchased from the Thermo Fisher Oxoid Co. Ltd., England (https://www.thermofisher.com). Agar was provided by the Biosharp Life Sciences Co. Ltd., China (http://www.biosharp.cn). The *E. coli* O157:H7 (CCTCC AB 200051) was obtained from the Center for Type Culture Collection, China (http://www.cctcc.org). All reagents used were of analytical grade and used without further processing.

### 4.2. Preparation of Growth Medium and Britton-Robison Buffer

Luria Bertani (LB) broth medium was prepared by dissolving 5 g yeast extract, 10 g peptone, and 5 g sodium chloride in one liter of distilled water. The lactose-selective medium was prepared by dissolving 25 g lactose, 15 g peptone, 1.5 g bovine bile salt, 0.08 g sodium carboxymethyl cellulose, 3 g sodium chloride, and 4 g dipotassium phosphate in one liter of distilled water. Both media were autoclaved at 121 °C and 15 psi for 25 min. For the preparation of LB agar plates, an additional 15 g/L agar was added to the LB broth medium. The Britton-Robison buffer was prepared by mixing 0.04 mol/L each of phosphoric acid, boric acid, and acetic acid, followed by the preparation of different pH solutions by adding 0.2 mol/L sodium hydroxide.

### 4.3. Synthesis and Characterization of CDs

The florescent CDs were synthesized through carbonization of sucrose with sufficient sulfuric acid, following the previously reported protocol [33] with some modifications (detailed in the Supplementary Materials), and characterized by various techniques. The fluorescence and absorption spectra of CDs were documented at room temperature by using the fluorescence spectrophotometer (RF-5301PC, Shimadzu, Japan) and Ultraviolet spectrophotometer (UV-2550, Shimadzu, Japan), respectively. The surface morphology and particle size of CDs were determined by transmission electron microscopy (TEM) (Tecnai G2 F30, FEI, Eindhoven, The Netherlands). The structural features of CDs were studied by an x’pert3 powder diffraction (XRD) instrument (PANalytical B.V., Eindhoven, The Netherlands), while the chemical properties were analyzed by using a Fourier transform infrared (FT-IR) spectrometer (VERTEX 70, Bruker, Germany).

The relationship between CDs and pH was measured by preparing a Britton-Robison buffer with different pH values followed by the addition of 1 mL CDs into 4 mL of Britton-Robison buffer. The fluorescence spectra of the mixture were recorded by using a fluorescence spectrophotometer at excitation wavelengths of 350 and 410 nm. The ratio of fluorescence values [I_F410_/I_F350_] was calculated at the maximum emission wavelength. Furthermore, the fluorescence stability of CDs was determined sporadically through the fluorescence spectrophotometer at excitation wavelengths of 350 and 410 nm for 70 days.

### 4.4. Bacterial Culture

The bacteria were cultured in the LB broth under shaking at 225 rpm for seven hours at 37 °C. The viable bacterial cells were counted by a classical colony count method and designated as colony-forming units per mL (CFU/mL). The bacterial culture was diluted to different dilutions, i.e., 13 CFU/mL, 67 CFU/mL, 1.33 × 10^2^ CFU/mL, 6.65 × 10^2^ CFU/mL, 1.33 × 10^3^ CFU/mL, 6.65 × 10^3^ CFU/mL, 1.33 × 10^4^ CFU/mL, 6.65 × 10^4^ CFU/mL, and 1.33 × 10^5^ CFU/mL, using the sterile normal saline. An aliquot of 10 μL of bacteria from different dilutions was transferred to a 10 mL lactose-selective medium and incubated at 37 °C for 13 h.

### 4.5. Detection of Bacteria

The pH of bacterial culture in the lactose-selective medium was determined via the pH-meter, and the linear relationship was plotted according to the pH of the medium and different bacterial cell density. For CDs-based fluorescent detection, a 4 mL bacterial culture in a lactose-selective medium was mixed with 1 mL of CDs (detailed in the Supplementary Materials). The fluorescence spectrum of the mixture was observed at excitation wavelengths of 350 and 410 nm, and the ratio of fluorescent intensity [I_F410_/I_F350_] was calculated at the maximum emission wavelength. The linear relationship was obtained according to the dilution of bacterial culture used and the ratio of fluorescence intensity of the mixture.

### 4.6. Detection of Bacteria in the Milk and Sewage Water

The practicability of synthesized CDs for bacterial detection was determined in the milk (purchased from the supermarket, Wuhan, China) and sewage water (obtained from Huxi stream, Wuhan, China). Before analysis, the sewage water was centrifuged at 4000 rpm for 10 min. Both milk and supernatant of sewage water were autoclaved at 121 °C for 25 min. For analysis, 100 μL of bacterial culture was added to 900 μL of milk and sewage water and analyzed by the plate count method. In parallel, 10 μL from both samples containing bacterial cells were separately inoculated into the 10 mL of lactose selective medium and incubated under shaking at 225 rpm and 37 °C for 13 h. Thereafter, 1 mL of CDs were added to 4 mL of each sample in the lactose-selective medium. The fluorescence spectra of both samples were recorded at excitation wavelengths of 350 and 410 nm for the detection of bacteria in the lactose-selective medium.

### 4.7. MALDI-TOF MS Analysis 

MALDI-TOF MS was carried out for the identification of the bacterial strain. For analysis, milk and sewage water were spiked with *E. coli*. In parallel, sterilized milk and sewage water were used as controls. All samples were analyzed by MALDI-TOF MS. The obtained data were processed using the microTyper MS (Jiangsu Skyray Instrument Co., Ltd., Kunshan, China). The results were imported to the database, and the specific bacterium was identified by comparing the ion mass and relative strength of each characteristic peak with the super and reference spectra in the standard spectrum database. The identification results were expressed in percentage, and each credibility level is distinguished by different colors.

## 5. Conclusions

The present study developed combination pH-sensitive fluorescent CDs and MALDI-TOF MS for the detection of *E. coli* O157:H7. The fluorescent CDs solution was synthesized simply through carbonization and demonstrated distinct properties, such as high hydrophilicity, pH sensitivity, and fluorescent stability. The developed CDs-based ratiometric pH probe and MALDI-TOF MS were effectively used to detect and identify bacteria with improved LOD (1 CFU/mL) compared to the magnetic CDs reported by Bhaisare et al. involving fluorescence followed by MALDI-MS [32]. The developed CDs-based probe detected *E. coli* even in real samples, like milk and sewage water, indicating their applicability for practical applications in bacterial detection. Thus, a combination of CDs-based ratiometric pH probes and MALDI-TOF MS can be potentially used in the food industry and for environmental monitoring.

## Figures and Tables

**Table 1:** *E. coli* O157:H7 detection in the milk and sewage water by CDs-based probe and agar plate count method.

Sample	Agar Plate Count Method (CFU/mL)	CDs Probe Method (CFU/mL)	Recovery (%)
Sewage water	66	67	101.5
Milk	13	12	92.3

**Table 2:** Comparison of the current work with the previously reported CDs-probe-based *E. coli* detection.

CDs Probe	Synthesis Route	Working Mechanism	Sample	LOD (CFU/mL)	Ref.
**Mannose-CDs**	Heating ammonium citrate at 180 °C for 2 h, further needed mannose modification	Combination of mannose and *E. coli*	Apple juice, human urine	100	[14]
**Colistin-CDs**	Heating diammonium hydrogen citrate and colistin sulphate at 180 °C for 1 h	Combination of colistin and *E. coli*	Human urine, apple juice	460	[20]
**Amikacin-CDs**	Heating diammonium hydrogen citrate and amikacin precursor at 180 °C for 4 h	Combination of amikacin and *E. coli*	Apple, orange juice	552	[44]
**Magnetic-CDs**	A 12 h reaction of acetic acid, chitosan and Fe_3_O_4_ NPs at 180 °C	Magnetic-CDs absorb on *E. coli*	PBS, urine	350	[32]
**pH-sensitive fluorescent CDs**	Simple carbonization of sucrose at room temperature for 30 min	Sensitivity of fluorescence intensity to varying pH	Milk, Sewage water	1	This work

## Supplementary Materials

Supplementary materials can be found at https://www.mdpi.com/2076-2607/8/1/53/s1.
