# Supplementary material for: Fluorimetric Detection of Single Pathogenic Bacterium in Milk and Sewage Water Using pH-Sensitive Fluorescent Carbon Dots and MALDI-TOF MS
Source: Microorganisms. 2019 Dec 26;8(1):53. doi: 10.3390/microorganisms8010053 (PMC7022441; doi:10.3390/microorganisms8010053)
Supplement: Supplementary file 1 [file microorganisms-08-00053-s001.pdf]

Synthesis and optimal fluorescence intensity of CDs at different pH values

The fluorescent CDs solution was prepared through carbonization of sucrose using concentrated sulfuric acid, followed by neutralization with saturated sodium hydroxide solution. In detail, 1 g sucrose was completely dissolved in deionized water (1:1 *w*/*v*) through vigorous stirring, followed by drop-wise addition of 1.2 mL of concentrated H_2SO_4 at room temperature to avoid the formation of precipitates during the reaction. Thereafter, 10 mL of distilled water was added to the reaction mixture and allowed to cool down at room temperature. The as-obtained homogeneous brown solution was neutralized by adding saturated NaOH solution. The CDs solution was stored at 4 °C for further analysis and use.

In order to obtain CDs with the highest fluorescence intensity under the same conditions, the pH was optimized during the synthesis process. For the synthesis of CDs at different pH, varying amounts of saturated NaOH were added, while keeping other experimental conditions constant. The fluorescence intensity of as-prepared CDs was measured at the maximum emission wavelength. As shown in Figure S1, the CDs produced at pH 5.68–7.49 demonstrated excellent fluorescence activity. The results also showed that the fluorescence intensity was highest at the pH range of 5–10, and lower at minimum and maximum pH values, i.e., <4 and >10, respectively. The lower fluorescent intensity at extreme pH could be attributed to their deteriorating effect towards fluorescent functional groups on the surface of CDs.

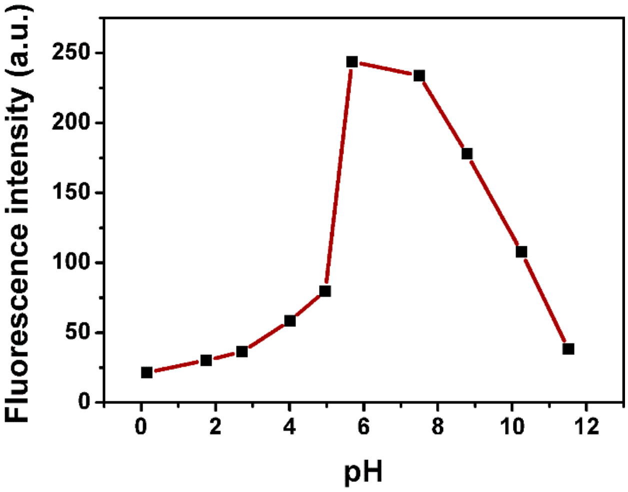

Figure S1. Effect of pH on the fluorescence intensity of CDs solutions, prepared at different pH by the addition of varying amounts of saturated NaOH.

Optimization of volume ration of CDs versus bacterial cell culture

For economical production, the optimal volume ratio of CDs to bacterial cell culture with the highest fluorescence intensity was determined. Briefly, 4 mL of bacterial cell culture in the lactose selective medium, was followed by the addition of different volumes of CDs, and their fluorescence intensity was observed at the maximum emission wavelength. The results are shown in Figure S2, showing a plot of the volume of CDs versus fluorescence intensity at the maximum emission wavelength. The determination of volume ratio of bacterial solution and CDs is essential from the economic perspective as it prevents the loss of CDs as it gives information about the maximum fluorescent activity. The results showed that the fluorescence intensity was increased with the increasing volume of CDs, from 0.1 to 1.2 mL. Figure S2 shows that the maximum fluorescence intensity was observed by the addition of 1.0 mL CDs solution to 4 mL of bacterial cell culture. Further addition of CDs solution to bacterial cell culture resulted in decreased fluorescent intensity. Thus, it can be concluded that the ratio between CDs solution and bacterial cell culture should be maintained at 1:4 for maximum fluorescence intensity.

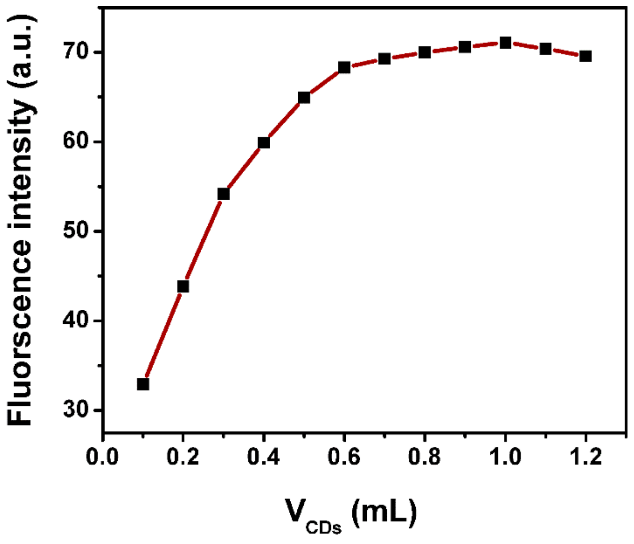

Figure S2. Optimization of volume ration of CDs versus bacterial cell culture. The maximum fluorescence intensity was achieved at 1:4 between CDs solution and bacterial cell culture in the lactose selective medium.
